# Intronic polymorphisms in genes *LRFN2* (rs2494938) and *DNAH11* (rs2285947) are prognostic indicators of esophageal squamous cell carcinoma

**DOI:** 10.1186/s12881-019-0796-9

**Published:** 2019-05-03

**Authors:** Jiru Wang, Qiuzi Wang, Bin Wei, Yu Zhou, Zhaoye Qian, Yong Gao, Xiaofei Chen

**Affiliations:** Department of Medical Oncology, The Affiliated Huaian No.1 People’s Hospital of Nanjing Medical University, Huai’an, 223300 People’s Republic of China

**Keywords:** Polymorphisms, rs2494938, rs2285947, Prognosis, Esophageal squamous cell carcinoma

## Abstract

**Background:**

Genome wide association study (GWAS) has become the major means to screen for the genetic variants associated with risk and prognosis of different diseases. A recent GWAS has discovered three novel intronic single nucleotide polymorphisms in genes *LRFN2* (rs2494938), *DNAH11* (rs2285947) and *PLCXD2* (rs2399395) that are associated with altered risk of esophageal squamous cell carcinoma (ESCC) among Han Chinese populations. However, the prognostic significance of these variations in ESCC remains unclear.

**Methods:**

To investigate the association of three novel single nucleotide polymorphisms (rs2494938, rs2285947, rs2399395) with the prognosis of ESCC patients, we recruited 287 ESCC patients treated with surgical resection and evaluated the potential significance of the three polymorphisms through Kaplan-Meier survival analysis, log-rank test, and Cox proportional hazards regression models.

**Results:**

The ESCC patients carrying genotype AA at rs2494938 had worse survival and genotype GG at 2285947 had better prognosis (Log-rank *P =* 0.003 and Log-rank *P =* 0.037, respectively). In addition, rs2494938 at 6p21.1 was independently associated with overall survival of ESCC patients in recessive model [AA vs. GG/GA, HR = 3.12, 95% CI = 1.43–6.83, *P* = 0.004], rs2285947 at 7p15.3 was independently associated with overall survival of ESCC patients in both dominant model [AA/GA vs. GG, HR = 1.59, 95% CI = 1.02–2.49, *P* = 0.042] and additive model [AA vs. GA vs. GG, HR = 1.45, 95% CI = 1.05–2.01, *P* = 0.025].

**Conclusions:**

This study demonstrated that the polymorphisms rs2494938 at 6p21.1 and rs2285947 at 7p15.3 may serve as independent prognostic biomarkers for ESCC, implying the potential biological role of their related genes (*LRFN2* and *DNAH11*) in the process of ESCC development.

**Electronic supplementary material:**

The online version of this article (10.1186/s12881-019-0796-9) contains supplementary material, which is available to authorized users.

## Background

Esophageal cancer is the sixth leading cause of cancer death among males and the ninth among females worldwide with the highest incidence in Eastern Asia, where the major histological subtype of esophageal cancer is esophageal squamous cell carcinomas (ESCC) [1]. Even with numerous investigations regarding the diagnosis and therapeutic strategies, the etiology of ESCC remains inconclusive and the clinical outcome remains poor.

As a powerful and universal strategy, genome-wide association studies (GWAS) has been used to uncover the susceptibility of complex diseases including malignancies [2, 3]. Nowadays, data generated by GWAS have expanded our understanding of genetic variants that also can act as prognostic markers for multiple cancers [4], such as the SNP rs10484761 in gastric cancer [5], SNP of *XRCC1* Arg399Gln both in non-small cell lung cancer [6] and breast cancer [7], the *GNAS1* T393C polymorphism in gastric cancer [8], laryngeal carcinoma [9] and breast cancer [10]. In the recent years, polymorphisms of several genes are expected to be potential markers for diagnosis and prognosis of ESCC, which will ultimately contribute to the development of novel therapeutic strategies for this disease. For example, polymorphism rs4919510 within *miR-608* was deemed to predict the survival of ESCC [11]. Another SNP C8092A at *ERCC1* showed an association with ESCC patients’ survival although the association is not statistically significant [12]. Additionally, Genome-wide association study identified several polymorphisms in *SLC39A6* that were associated with length of survival in ESCC [13].

Recently, Jin et al. performed a GWAS study and discovered that three novel intronic susceptibility loci in the genes *LRFN2* (rs2494938 at 6p21.1), *DNAH11* (rs2285947 at 7p15.3) and *PLCXD2* (rs2399395 at 3q13.2) were associated with the risk of ESCC in Han Chinese populations [14]. These findings highlight the important roles of these genetic alterations in ESCC. In present study, we further explored the association between three SNPs and overall survival in ESCC and discovered that rs2494938 in *LRFN2* and rs2285947 in *DNAH11* might emerge as potential prognostic factors of ESCC in a Chinese population.

## Methods

### Patients samples

Our study was approved by the institutional review board of the Affiliated Huaian No.1 People’s Hospital of Nanjing Medical University. A total of 287 ESCC cases were recruited from the Affiliated Huaian No.1 People’s Hospital of Nanjing Medical University with their surgical resections between 2006 and 2010. All participants were ethnic Han Chinese and histopathologically diagnosed as ESCC. The clinical features of ESCC patients including sex, age, tumor-node-metastasis (TNM) stage, histologic grade, as well as history of smoking and drinking were gathered from medical records. The TNM stage was based on the 8th edition of the American Joint Commission for Cancer Staging (AJCCS) classification. Individuals who smoked > 1 cigarette every day for at least one year were defined as smokers, and the others were classified as nonsmokers. Drinkers were defined if individuals drink no less than twice a week for at least one year, and the others were defined as nondrinkers.

Approval for the use of patient samples and clinical characteristics was obtained from the Ethics Committee of The Affiliated Huaian No.1 People’s Hospital of Nanjing Medical University and the experiments in this study were conducted with all patients’ written informed consent.

### Genotyping

Approximately 5 ml peripheral vein blood was collected from each recruited subject before the surgery. Leukocyte genomic DNA was extracted by the QIAamp® DNA Mini-Kit (Qiagen, San Diego, CA) according to the manufacturer’s protocol. TaqMan allelic discrimination assays were performed on the ABI 7900 genotyping platform (Applied Biosystems, Foster City, CA, USA) for the SNP genotyping. The basic information of the selected SNPs and details of the corresponding primers and probes used were shown in the Additional file 1: Tables S1-S2. The genotypes were analyzed by using the SDS 2.3 Allelic Discrimination Software (Applied Biosystems, Foster City, CA, USA). Laboratory personnel were blind to the subjects’ information and performed the genotyping independently.

### Statistical analysis

All data were statistically analyzed with SPSS version 19.0 software package (IBM, Armonk, NY, USA). Kaplan-Meier survival curve was used to evaluate the survival probabilities and log-rank test was used to analyze the significance differences. Multivariate or univariate Cox regression analysis was used to determine predictive factors of ESCC survival by estimating the hazard ratios (HRs) and their 95% confidence intervals (CIs) with adjustment for age, gender, tumor site, TNM stage, tumor differentiation, and smoking and drinking status. *P* < 0.05 was considered to indicate statistical significance.

## Results

### Clinical characteristics of ESCC patients

The demographic characteristics and clinical features of 287 patients with ESCC were summarized in Table 1. The median age was 61 years (range from 40 to 83 years), and there were 187 (65.2%) males and 100 (34.8%) females. Among them, 88 patients had TNM stage III tumors at the time after surgery. The median follow-up time is 54 months (range from 1.1 to 67.9 months). During the entire follow-up period, 82 (28.6%) patients died of ESCC. The results of univariate Cox regression analyses revealed no statistically significant impact of age, tumor site, tumor differentiation and drinking status on overall survival of ESCC patients. However, gender, smoking status, and TNM stage showed a significant association with ESCC patients’ survival (Log-rank *P* < 0.05). Compared with male individuals, female patients had a significant lower risk of death (HR = 0.52, 95% CI = 0.33–0.82, Log-rank *P* = 0.005). Meanwhile, smoking patients had higher risk of death than no-smoking counterparts (HR = 1.57, 95% CI = 1.01–2.44, Log-rank *P* = 0.046). As the TNM stage increased, the risk of death exhibited a significant increase (Log-rank *P* < 0.001).

**Table 1:** Univariate analysis of clinical characteristics associated with post-operational overall survival in ESCC patients

Characteristics	Patients	Deaths	HR (95% CI)	Log-rank *P*
	*n* = 287 (%)	*n* = 82 (%)		
Age (years)				
≦ 61	150 (52.3)	46 (30.7)	1	0.502
> 61	137 (47.7)	36 (26.3)	0.858 (0.56–1.33)	
Gender				
Male	187 (65.2)	64 (34.2)	1	**0.005**
Female	100 (34.8)	18 (18.0)	**0.52 (0.33–0.82)**	
Tumor site				
Upper	16 (5.6)	6 (37.5)	1	0.488
Middle	252 (87.8)	72 (28.6)	0.675 (0.29–1.55)	
Lower	19 (6.6)	4 (21.1)	0.475 (0.13–1.68)	
Tumor differentiation				
G1	54 (18.8)	10 (18.5)	1	0.101
G2/G3	233 (81.2)	72 (30.9)	1.58 (0.91–2.74)	
TNM stage				
I	31 (10.8)	5 (16.1)	1	**< 0.001**
II	199 (69.3)	46 (23.1)	1.52 (0.61–3.83)	
III	57 (19.9)	31 (54.4)	**2.19 (1.36–3.52)**	
Smoking status				
No	170 (59.2)	40 (23.5)	1	**0.046**
Yes	117 (40.8)	42 (35.9)	**1.57 (1.01–2.44)**	
Drinking status				
No	198 (69.0)	55 (27.8)	1	0.967
Yes	89 (31.0)	27 (30.3)	1.01 (0.64–1.60)	

Abbreviations: *ESCC* esophageal squamous cell carcinoma, *HR* hazard ratio, *CI* confidence interval, *TNM* tumor node metastasis

Log-rank *P* and HR value were marked bold when it was with a significant level of Log-rank *P* < 0.05

### Association of rs2494938, rs2285947 and rs2399395 with ESCC prognosis

**Fig. 1:**
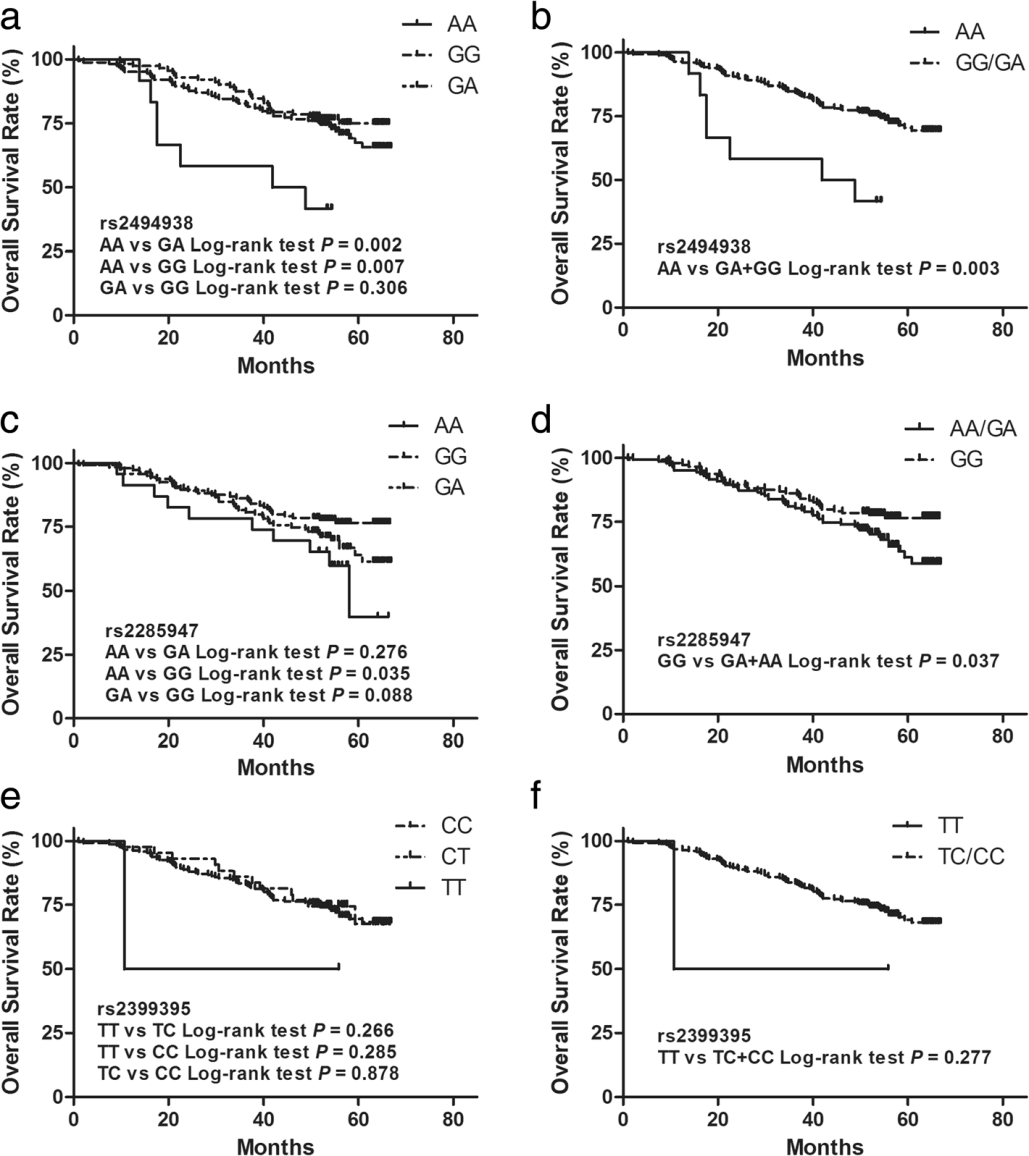
Kaplan-Meier survival curves for ESCC patients with the different genotypes of rs2494938 ((**a**, **b**) 163 events for GG genotype, 112 events for GA genotype, 12 events for AA genotype), rs2285947 ((**c**, **d**) 145 events for GG genotype, 119 events for GA genotype, 23 events for AA genotype), rs2399395 ((**e**, **f**) 242 events for CC genotype, 43 events for TC genotype, 2 events for TT genotype) polymorphisms; SNP rs2494938 and rs2285947 was correlated with the overall survival in ESCC patients (**a**-**d**), but not rs2399395 (**e**, **f**)

Kaplan-Meier curves were used to assess the association of patients’ survival with polymorphisms rs2494938, rs2285947 and rs2399395 (Fig. 1). Significant difference in survival rate was discovered in ESCC patients with different genotypes at rs2494938 (Fig. 1a, Fig. 1b) and rs2285947 (Fig. 1c, Fig. 1d), but not rs2399395 (Fig. 1e, Fig. 1f). Kaplan-Meier survival curves and log-rank test indicated that ESCC patients harboring genotype AA at rs2494938 suffered a shorter survival time than those with genotype GG and GA alone (Fig. 1a, Log-rank *P* = 0.007, Log-rank *P* = 0.002, respectively). Consistently, ESCC patients carrying G allele (GG + GA) at rs2494938 had better prognosis (Fig. 1b, Log-rank *P* = 0.003). What’s more, ESCC patients harboring genotype AA at rs2285947 had shorter survival time than those with genotype GG (Fig. 1c, Log-rank *P* = 0.035) and the patients carrying A allele (AA + GA) at rs2285947 had worse survival (Fig. 1d, Log-rank *P* = 0.037). The above suggested that A allele of rs2494938 or rs2285947 was an independent risk factor for overall survival in ESCC patients.

In addition, the association of three SNPs with survival time in ESCC was analyzed by univariate or multivariate cox regression analysis under different genetic models (additive model, dominant model and recessive model) and presented in Table 2. SNP rs2494938 was consistently demonstrated to be independently associated with overall survival in recessive model (HR = 3.12, 95% CI = 1.43–6.83, *P* = 0.004), so was rs2285947 in both dominant model (HR = 1.59, 95% CI = 1.02–2.49, *P* = 0.042) and additive model (HR = 1.45, 95% CI = 1.05–2.01, *P* = 0.025). However, no correlation between SNP rs2399395 and overall survival was demonstrated in different genetic models (*P* > 0.05, Table 2).

**Table 2:** Genotyping results with ESCC patients’ survival

SNPs	Patients^a^	Deaths^a^	Genetic models	Univariate analysis		Multivariate analysis	
				HR (95% CI)	*P*	HR (95% CI)^e^	*P* ^e^
rs2494938	163/112/12	48/27/7	Additive^b^	1.12 (0.76–1.64)	0.566	1.14 (0.78–1.67)	0.509
			Dominant^c^	0.92 (0.59–1.43)	0.705	0.94 (0.60–1.46)	0.776
			Recessive^d^	**3.10 (1.42–6.75)**	**0.004**	**3.12 (1.43–6.83)**	**0.004**
rs2285947	145/119/23	33/38/10	Additive^b^	**1.48 (1.07–2.04)**	**0.018**	**1.45 (1.05–2.01)**	**0.025**
			Dominant^c^	**1.60 (1.03–2.48)**	**0.038**	**1.59 (1.02–2.49)**	**0.042**
			Recessive^d^	1.77 (0.91–3.43)	0.092	1.68 (0.85–3.31)	0.135
rs2399395	242/43/2	69/12/1	Additive^b^	1.06 (0.61–1.85)	0.841	0.95 (0.54–1.66)	0.851
			Dominant^c^	1.00 (0.56–1.82)	0.989	0.90 (0.50–1.65)	0.736
			Recessive^d^	2.85 (0.40–20.48)	0.299	1.90 (0.26–14.01)	0.530

Abbreviations: *CI* confidence interval, *HR* hazard ratio

Values in bold indicate they are statistically different (*P* < 0.05)

^a^Wild homozygous type / Heterozygote / Variant homozygous type

^b^Rare homozygote versus heterozygote versus major homozygote

^c^Heterozygote/rare homozygote versus major homozygote

^d^Rare homozygote versus heterozygote/major homozygote

^e^Adjusted for age, gender, tumor site, TNM stage, tumor differentiation, smoking and drinking status in Cox regression model

## Discussion

In the present study, we evaluated the SNPs (rs2494938, rs2285947 and rs2399395) which were potentially involved in the carcinogenesis of ESCC, and identified three genetic variations associated with prognosis of ESCC patients in Chinese populations. Herein, for the first time, we demonstrated the prognostic significance of rs2494938 and rs2285947 in ESCC.

In this study, the AA homozygous genotype of SNP rs2494938 in *LRFN2* was significantly associated with worse survival outcome of ESCC. Genetic variants at 6p21.1, where SNP rs2494938 locates and its amplification has been frequently detected in human cancers [15], have been discovered as independent prognostic markers for cancers, such as SNP rs10484761 for gastric cancer and SNP rs2494938 for laryngeal carcinoma [5, 16]. The rs2494938 is located in the intron region of *LRFN2* (Leucine-rich repeat and fibronectin type III domain-containing protein 2). The protein of LRFN2, as a component of membrane, has been found to interact with N-methyl-D-aspartate receptors (NMDARs) to participate in the neuron development and synapse function [17, 18]. Furthermore, several studies indicated that NMDARs played important roles in the development and progression of multiple cancers, including non-small cell lung carcinoma, gastric cancer, colorectal carcinoma and ovarian cancer [19–23]. Also, aberrant methylation of the NMDAR2B abrogated gene transcription leading to cellular resistance to apoptosis, which was strongly related to clinical outcomes of ESCC [24, 25]. All these support that targeting NMDARs serves potentially as a therapeutic strategy. However, whether the locus at 6p21.1 could function as a prognostic gene for ESCC through LRFN2-NMDAR pathway is largely unknown.

Until now, evidences have confirmed the association of the polymorphism rs2285947 in *DNAH11* with the higher risk of human cancers, such as ovarian cancer, head and neck cancer, lung cancer, non-cardia gastric cancer and esophageal squamous cell carcinoma [14, 26, 27]. We found that the GA/AA genotype of rs2285947 exhibited a significant association with poorer survival than the GG genotype among ESCC patients. The rs2285947 at 7p15.3 is located in the intron region of *DNAH11* (Dynein, axonemal, heavy chain11) which encodes a ciliary outer dynein arm protein and is a member of the dynein heavy chain family. As a member of microtubule-associated motor protein complexes, dynein plays an indispensable role on activation of MAPK (mitogen-activated protein kinase) kinase 3/6 and p38 to regulate multiple crucial biological progresses in vivo, such as cell survival, differentiation and migration, as well as in the inflammatory and immune response [28–32]. Thus, the polymorphism rs2285947 may play an important role on the expression of gene *DNAH11*.

Based upon our study, further functional investigations are warranted to figure out the potential biological importance of the cancer prognosis-related locus of rs2494938 and rs2285947 in ESCC. Nevertheless, there are two major limitations in this study. First, the sample size of this research was small, particularly in subgroup carrying AA genotype of rs2494938 and TT genotype of rs2399395. Second, this study recruited patients only from one region and the data was not validated in an independent group, which might lead to potential selection bias. Cautions should be utilized when the results are applied to other populations.

## Conclusions

Taken together, we discovered that rs2494938 at 6p21.1 and rs2285947 at 7p15.3 may play important roles in the survival in ESCC patients. The present study confirmed that the polymorphisms rs2494938 in *LRFN2* and rs2285947 in *DNAH11* may become independent prognostic markers for ESCC in Chinese population. Further investigation is being undertaken to uncover the molecular mechanisms of these intronic genetic variants and the function of their related genes in the development of ESCC.

## Additional file

Additional file 1: **Table S1.** The basic information of the selected SNPs. **Table S2.** Information of Primers and Probes for TaqMan Allelic Discrimination. (DOCX 18 kb)

## Abbreviations

DNAH11: Dynein, axonemal, heavy chain11
ESCC: Esophageal squamous cell carcinoma
GWAS: Genome wide association study
LRFN2: Leucine-rich repeat and fibronectin type III domain-containing protein 2
MAPK: Mitogen-activated protein kinase
NMDAR: N-methyl-D-aspartate receptor
SNP: Single nucleotide polymorphism
