# Supplementary material for: Intronic polymorphisms in genes LRFN2 (rs2494938) and DNAH11 (rs2285947) are prognostic indicators of esophageal squamous cell carcinoma
Source: BMC Med Genet. 2019 May 3;20:72. doi: 10.1186/s12881-019-0796-9 (PMC6499982; doi:10.1186/s12881-019-0796-9)
Supplement: Supplementary file 1 — Table S1. The basic information of the selected SNPs. Table S2. Information of Primers and Probes for TaqMan Allelic Discrimination. (DOCX 18 kb) [file 12881_2019_796_MOESM1_ESM.docx]

**Additional file 1**

**Table S1** The basic information of the selected SNPs

| SNPs | chromosome | genes | location | Allele changes^a^ | MAF |
| --- | --- | --- | --- | --- | --- |
| rs2494938 | 6p21.1 | *LRFN2* | intron | G＞A | 0.262 |
| rs2285947 | 7p15.3 | *DNAH11* | intron | G＞A | 0.292 |
| rs2399395 | 3q13.2 | *PLCXD2* | intron | C＞T | 0.095 |

MAF, minor allele frequency.

^a^Allele changes, major > minor.

**Table S2** Information of Primers and Probes for TaqMan Allelic Discrimination

| SNPs |  | Sequence (5'-3') |
| --- | --- | --- |
| rs2494938 | Primers | F: CCTGGGATCTATGTGTCTAAGATGG |
|  |  | R: TGTAAGCCACCCACATACATAACTT |
|  | Probes | G: FAM-AGACAGCAGCTTC-MGB |
|  |  | A: HEX-AGACAGCAACTTC-MGB |
| rs2285947 | Primers | F: GGCTGCCTTATGGTATTGTGAATTT |
|  |  | R: ACAACTACTGAGTCATTCCATTGAACAT |
|  | Probes | G: FAM-ACAAGGCGATAGAAC-MGB |
|  |  | A: HEX-ACAAGGCAATAGAAC-MGB |
| rs2399395 | Primers | F: TCAAATACTTCCTCAGGGCAGAA |
|  |  | R: GGAACACCATCTGATACAAAATTACTG |
|  | Probes | T: FAM-TGAGCCTGGTTGTAC-MGB |
|  |  | C: HEX-TGAGCCTGGCTGTAC-MGB |
